# FANCI serve as a prognostic biomarker correlated with immune infiltrates in skin cutaneous melanoma

**DOI:** 10.3389/fimmu.2023.1295831

**Published:** 2023-11-22

**Authors:** Zhenguo Cai, Yanjuan Duan, Wen Li, Zhuohang Liu, Zijun Gong, Sheng Hong, Xu He, Xinyang Xuanyuan, Youdong Chen, Xinling Bi, Wuqing Wang

**Affiliations:** ^1^ Department of Dermatology, Shuguang Hospital, Shanghai University of Traditional Chinese Medicine, Shanghai, China; ^2^ Department of Dermatology, Changhai Hospital, Naval Medical University, Shanghai, China; ^3^ Department of Dermatology, Seventh People’s Hospital of Shanghai University of Traditional Chinese Medicine, Shanghai, China; ^4^ Department of Neurology, Minhang Hospital, Fudan University/Central Hospital of Minhang District, Shanghai, China; ^5^ Department of General Surgery, Zhongshan Hospital, Fudan University, Shanghai, China; ^6^ Department of Dermatology, Huashan Hospital, Fudan University, Shanghai, China

**Keywords:** FANCI, skin cutaneous melanoma, prognostic biomarker, immune infiltrates, immunotherapy

## Abstract

**Background:**

As a member of tumor, Skin cutaneous melanoma (SKCM) poses a serious threat to people’s health because of its strong malignancy. Unfortunately, effective treatment methods for SKCM remain lacking. FANCI plays a vital role in the occurrence and metastasis of various tumor types. However, its regulatory role in SKCM is unclear. The purpose of this study was to explore the association of FANCI with SKCM.

**Methods:**

This study investigated the expression of FANCI in GSE46517, GSE15605, and GSE114445 from the Gene Expression Omnibus database and The Cancer Genome Atlas (TCGA)-SKCM datasets using the package “limma” or “DESeq2” in R environment and also investigated the prognostic significance of FANCI by utilizing the GEPIA database. Additionally, our research made use of real-time quantitative polymerase chain reaction (RT-qPCR) and immunohistochemical (IHC) staining to verify FANCI expression between SKCM and normal tissues and developed the knockdown of FANCI in A375 and A875 cells to further analyze the function of FANCI. Finally, this study analyzed the correlation of FANCI and tumor-infiltrating immune cells by CIBERSORT, ESTIMATE, and ssGSEA algorithms.

**Results:**

The FANCI level was increasing in SKCM tissues from GSE46517, GSE15605, GSE114445, and TCGA-SKCM. However, high FANCI expression correlated with poor overall survival. The RT-qPCR and IHC confirmed the accuracy of bioinformatics. Knocking down FANCI suppresses A375 and A875 cell proliferation, migration, and invasion. FANCI could be involved in the immunological milieu of SKCM by regulating immune responses and infiltrating numerous immune cells, particularly neutrophils, CD8+ T cells, and B cells. Furthermore, patients with SKCM who have a high FANCI expression level are reported to exhibit immunosuppression, whereas those with a low FANCI expression level are more likely to experience positive outcomes from immunotherapy.

**Conclusions:**

The increased FANCI expression in SKCM can be a prognostic biomarker. Knockdown FANCI can reduce the occurrence and progression of SKCM. The FANCI expression provides a foundation for predicting the immune status and treatment of SKCM.

## 1 Introduction

Skin cutaneous melanoma (SKCM) is the most pernicious type of skin cancer resulted from melanocytes (1) and is mainly prevalent in the head and neck (2). The pathogenesis is unknown, but potential factors include genetic predisposition and a history of melanoma, specifically sun exposure (3). The epidemiology shows an increasing SKCM incidence and prevalence, with approximately 192,000 new SKCM diagnoses in the United States annually (4) and an approximately 17% mortality rate for patients with SKCM in 2020 (5). Early diagnosis is crucial for treating SKCM, and surgical resection remains an ideal treatment modality for most cases with early-stage SKCM (6). Most SKCM patients are only detected at an advanced stage when the patient’s prognosis is significantly worse (7). The overall survival (OS) of SKCM patients has been increased with immunotherapy and targeted therapy, but different drug efficacy due to individual differences causes different prognoses and is very prone to drug resistance (8). Therefore, researchers should develop effective and new biomarkers to improve the diagnostic value of SKCM and personalize patient treatments.

Fanconi anemia (FA) is defined by anemia and bone marrow failure (9). As one of 22 FA family proteins, FANCI functions as a significant role in FA occurrence. FANCI and FANCD2 are in a central position of the FA signaling (10). FANCI has important functions at other cell cycle stages, directly through integration into other pathways, in addition to its important role in the FANCD2-FANCI of DNA damage or binding to ataxia telangiectasia mutated and Rad3-related (ATR) during DNA replication (11, 12). FANCI-/- mice exhibit typical FA features, such as microphthalmia, and blood system malfunction (13). Additionally, the occurrence of multiple tumors involved FANCI. FANCI expression was upregulated in cervical cancer, and upregulated FANCI predicts poor prognosis in patients (14). FANCI expression level was higher in non−small cell lung cancer tumor tissues, and the FANCI knockdown inhibited the progression by activating EMT *in vivo* and *in vitro* (15). The occurrence of familial ovarian cancer may involve the missense variant of FANCI (16). Akt is a critical mediator in cancer, and FANCI negatively regulates AKT activation (17). Up to present, the key role of FANCI in SKCM tumorigenesis and the specific mechanism involved remains unknown.

This study investigated FANCI expression in SKCM tissues by SKCM-The Cancer Genome Atlas (TCGA) and Gene Expression Omnibus (GEO) datasets. We first verified the accuracy of bioinformatics through IHC and real-time RT-qPCR. Our study revealed that patients with SKCM with higher FANCI levels had a worse prognosis. Next, we developed the FANCI knockdown in A375 and A875 cells. The knocking down of FANCI suppressed the proliferation, migration, and invasion abilities of SKCM. Finally, we investigated the association between FANCI and immune cells. This study, for the first time, clarified the correlation between FANCI and SKCM. Therefore, FANCI will be a promising biomarker that predicts the clinical outcomes and immune therapy measure for treating patients with SKCM.

## 2 Materials and methods

### 2.1 Data acquisition and mRNA expression analysis

The RNA sequence datasets were downloaded from the public GEO and TCGA) databases. We analyzed GSE46517, GSE15605, GSE114445, and TCGA-SKCM datasets using the package “limma” (18) or “DESeq2” (19) in R environment to explore the mRNA expression of the overall differentially expressed genes (DEGs) between tumors, including primary and metastatic, and non-tumor tissues.

### 2.2 Prognostic analysis

We exploited the GEPIA (http://gepia.cancer-pku.cn/) database (20) to probe into the relationship between FANCI and OS, in which we used the median FANCI gene expression as a cut-off value to categorize the groups.

### 2.3 Immune infiltration cells analysis

This study explored the relevance between the FANCI expression level and related immune infiltration in SKCM using the TIMER database (21, 22) and analyzed Kaplan–Meier plots (K-M) of immune cell infiltration and FANCI expression levels in SKCM. Additionally, this study explored the correlations between FANCI expression and the abundance of tumor-infiltrating lymphocytes (TILs) of FANCI via the “Lymphocyte” module in TISIDB (http://cis.hku.hk/TISIDB/index.php) (23).

This study exploited the CIBERSORT algorithm (24) to assess the abundance of 22 immune cells in the higher and lower FANCI expression classifications within the TCGA-SKCM cohort applying clinical bioinformatics assistance. Additionally, this study analyzed the correlation of FANCI and 22 kinds of immune cells using the Spearman algorithm.

We made use of the “estimate” R package to determine the immune purity of the TCGA-SKCM expression matrix and examine the effect of FANCI on the immune microenvironment of SKCM. For each sample, we used the ssGSEA method and assessed immune infiltration through the ESTIMATE algorithm, which provided us with ImmuneScore, StromalScore, EstimateScore, and TumorPurity (25).

We applied the “GSVA” R package to process the normalized SKCM-TCGA data, as well as ssGSEA to classify gene sets based on their common biological functions, chromosomal location, and physiological regulation. The gene set consists of 782 genes, which predicts the abundance of 28 tumor-infiltrating immune cells (TIICs) in individual tissue samples (26). We demonstrate the enrichment of these 28 TIICs in both the high and the low FANCI expression groups by comparing the normalized SKCM-TCGA data with the gene set.

### 2.4 Immunotherapy responses analysis

The study examined the expression of eight classic immune checkpoint genes (PDCD1, LGALS9, cytotoxic T lymphocyte (CTL)A4, CD70, CD27, TNFRSF18, CCL5, and SELP) in different FANCI groups to investigate the sensitivity of patients in the high and low FANCI groups to immunotherapy. The study obtained the correlation between FANCI and immune checkpoint genes through the Spearman algorithm. We used the median gene expression of immune checkpoint genes as a cut-off value to investigate the relationship between immune checkpoint genes and OS in SKCM.

### 2.5 Immunohistochemical staining

Tissues contain 20 samples of SKCM tissues (n = 10) and para-cancerous tissues (n = 10) from Shanghai Shuguang Hospital. Briefly, the slices were first deparaffinized, and then pressure cooked for 25 min in EDTA antigen retrieval buffer (pH = 8.0, ZSGB-BIO, China). Additionally, we inhibited the endogenous peroxidase activity in 0.3% H2O2. After washing with phosphate-buffered saline (PBS), the slices were cultured with the primary antibody (1:200) overnight at 4°C in a humidified box. After 24 h, the secondary antibody was introduced, and these sections were treated at 37°C for 60 min. Lastly, we used the peroxidase and 3,3′-diaminobenzidine tetrahydrochloride for visualization. We categorized these average values of immune reactivity into five scoring groups: 1, not detected; 2, <10% positive cells; 3, 10%–20% weak to moderate positive cells; 4, 10%–20% strong positive cells or 20%–50% weak positive cells; 5, 20%–50% positive cells with moderate to significant reactivity or >50% positive cells. The antibodies used in the experiments were all purchased from Proteintech.

### 2.6 Real-time quantitative PCR

We used Trizol (Thermo Fisher Scientific, USA) to harvest the total cellular RNA or tissue RNA, which was then used for reverse transcription. We synthesized the cDNA using HisScript@ III RT SuperMix kit (Vazyme, China). The reference gene included glyceraldehyde-3-phosphate dehydrogenase (GAPDH). We repeated all the experiments three times. **Table 1** lists the primer sequences.

**Table 1:** Specific primers for RT-qPCR.

Gene	Forward (5’→3’)	Reverse (5’→3’)
*FANCI*	CCACCTTTGGTCTATCAGCTTC	CAACATCCAATAGCTCGTCACC
*CD115*	GGGAATCCCAGTGATAGAGCC	TTGGAAGGTAGCGTTGTTGGT
*CCR8*	GTGTGACAACAGTGACCGACT	CTTCTTGCAGACCACAAGGAC
*IL-1α*	TGGTAGTAGCAACCAACGGGA	ACTTTGATTGAGGGCGTCATTC
*TGFB1*	GGCCAGATCCTGTCCAAGC	GTGGGTTTCCACCATTAGCAC
*GAPDH*	ACTCCCATTCTTCCACCTTTG	CCCTGTTGCTGTAGCCATATT

### 2.7 Cell culture

We cultured A375 and A875 cells in Dulbecco’s Modified Eagle Medium (Gibco, USA) with 10% fetal bovine serum (ExCell Bio, China) and 100 U/ml penicillin–streptomycin (Seven, China) at 37°C with 5% CO2. Cells were carefully processed to prevent contamination and were identified through short tandem repeat profiling to ensure their quality.

### 2.8 Establishment of FANCI knockout A375 or A875 cells

We used the pLKO.1 short hairpin RNA (shRNA) lentivirus system from Ruibo Biotechnology Co., Ltd. (Guangzhou, China) to generate shRNA against human FANCI gene. In brief, we seeded A375 or A875 cells to be 70%–90% confluent at transfection. Afterward, we combined 5 µl of Lipofectamine 2000 reagent (Invitrogen, USA) and 10 µl of shRNA-FANCI-1 or shRNA-FANCI-2 (10 mM) in 500 µl of Opti-MEM (Invitrogen, USA) culture medium to incubate at room temperature for 30 min. We then added the mixture into a culture dish that already contained 2 ml of culture medium. We obtained shRNA-FANCI-1 or shRNA-FANCI-2 cells by puromycine (5 μg/ml) selection.

### 2.9 Western blot

We lysed the cells and used the protein kit (Fdbio, Hangzhou, China) to detect the specific concentration of protein. We then isolated and transferred the proteins to a polyvinylidene difluoride membrane (Millipore, Burlington, MA, USA), and then blocked with prepared 5% skim milk for 120 min and overnight at 4°C with primary antibody. We then treated the membranes with a secondary antibody conjugated with HRP (1:4000, Fdbio, Hangzhou, China) at ordinary temperature for 60 min. This study used anti-FANCI (1:1000, 67304-1-Ig, Proteintech) and GAPDH (1:5000, 60004-1-Ig, Proteintech) as antibodies.

### 2.10 5-ethynyl-2-deoxyuridine analysis

We spread cells evenly in well plates at a density of 1 × 10^5^ per well for further detection. We used the EdU reagent kit (Beyotime, China) to assay the proliferation ability of various cells, following the manufacturer’s instructions. We used fluorescence microscopy to observe the images.

### 2.11 Cell scratch assay

We seeded the cells to be 70%–90% confluent in a six-well plate. We used a sterile 200-μl pipette tip to create a concentrated scratch area in the cell culture medium. Afterward, we cultured the cells in a complete medium for 24 h. At different intervals, we observe the cells’ movement toward the damaged surface applying a microscope.

### 2.12 Cell invasion analysis

We measured the cell invasion ability using a Transwell plate (Corning, USA) with an 8-mm pore polycarbonate membrane. In brief, we seeded 5 × 10^5^ cells in serum-free suspension in the top chamber of the matrix gel-coated membrane. After no less than 24h of incubation, we continued the fixation and subsequently stained the cells on the underside of the membrane using crystal violet.

### 2.13 Apoptosis assays

We placed cells from different groups in the logarithmic growth phase in 6-well plates, treated them with EDTA-free trypsin, rinsed them with PBS, and collected them. We then resuspended the cells in 100 μL of binding buffer, then added 5 μL of Annexin V staining solution and 10 μL of PI staining solution. We vigorously mixed the cells using a pipette and left them at room temperature for 15 min. Before loading the machine, we added 400 μL of binding buffer solution to each sole tube and thoroughly mixed them, which resulted in a system volume of 500 μL. We analyzed the apoptosis of cells in each group using flow cytometry.

### 2.14 Statistical analysis

We made use of GraphPad Prism 8.0 and R 4.0.2 software for analyzing statistical significance of experimental data. We presented measurement data as mean ± standard deviation and used Student’s t-test and Dunnett’s T3 to analyze the data, with P-values of <0.05 indicating significance. *P < 0.05, **P < 0.01, ***P < 0.001.

## 3 Results

### 3.1 FANCI is significantly upregulated in SKCM

**Figure 1:**
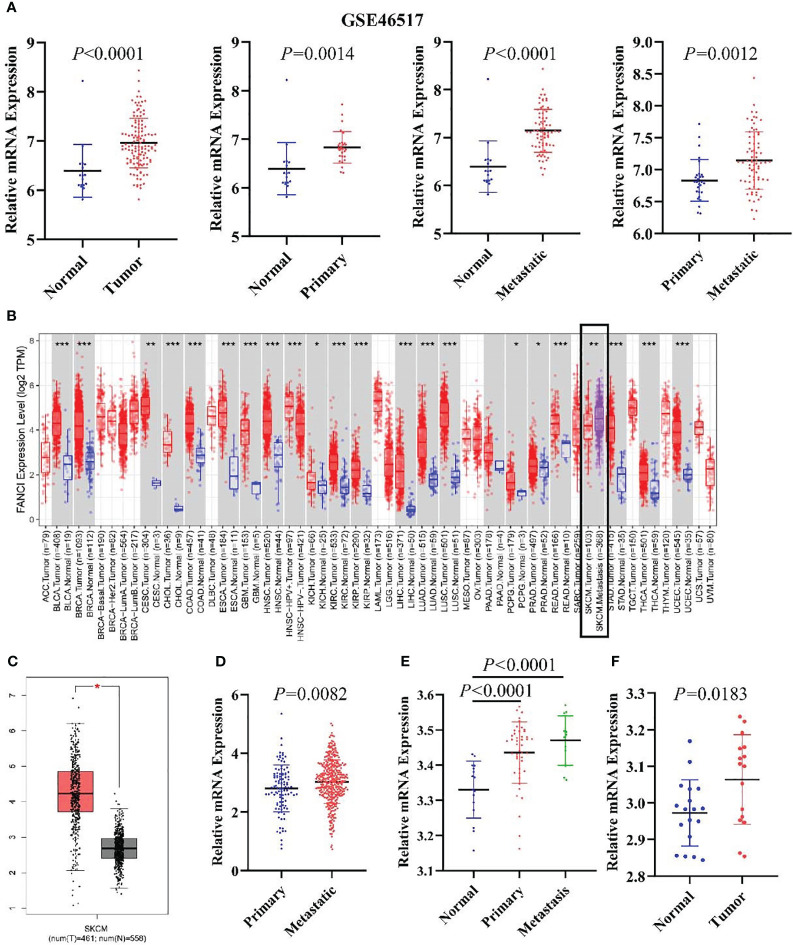
FANCI is significantly upregulated in SKCM. **(A)** The FANCI expression level in the normal, primary, and metastatic tumor tissues of SKCM from GSE46517. **(B)** Differential FANCI expression levels between tumor and adjacent normal tissues in the TIMER datasets. **(C)** FANCI was increased in SKCM from the GEPIA database. **(D)** FANCI was increased in SKCM metastatic tumors from TCGA. **(E)** FANCI expression in normal, primary, and metastatic tumor tissues of SKCM from the GSE15605 dataset. **(F)** FANCI was increased in SKCM from the GSE114445 dataset. *P < 0.05, **P < 0.01, ***P < 0.001.

We identified DEGs between SKCM and normal tissues in the microarray data GSE46517 dataset and applied the criteria of Log_2_|FoldChange>1| and P < 0.05. We screened the 1829 DEGs, including 1079 downregulated genes and 750 upregulated genes (SKCM versus healthy skin tissue) (**Supplementary Figures 1A, B**). FANCI was significantly upregulated in both primary and metastatic SKCM tissues (**Figure 1A**). Additionally, FANCI expression of metastatic tumor tissues exceeded that of tumor tissues in the GSE46517 dataset and TCGA-SKCM (**Figures 1A, B, D**). Consistently, FANCI expression was increased in SKCM tissues from the GEPIA dataset (**Figure 1C**). Furthermore, FANCI expression increased in the SKCM tissue versus the healthy skin tissue from GSE15605 (**Figure 1E**) and GSE114445 (**Figure 1F**).

FANCI expression of metastatic tumor tissues exceeded that of tumor tissues in the GSE46517 dataset and TCGA-SKCM.

### 3.2 Prognostic value of FANCI in multiple cancers

**Figure 2:**
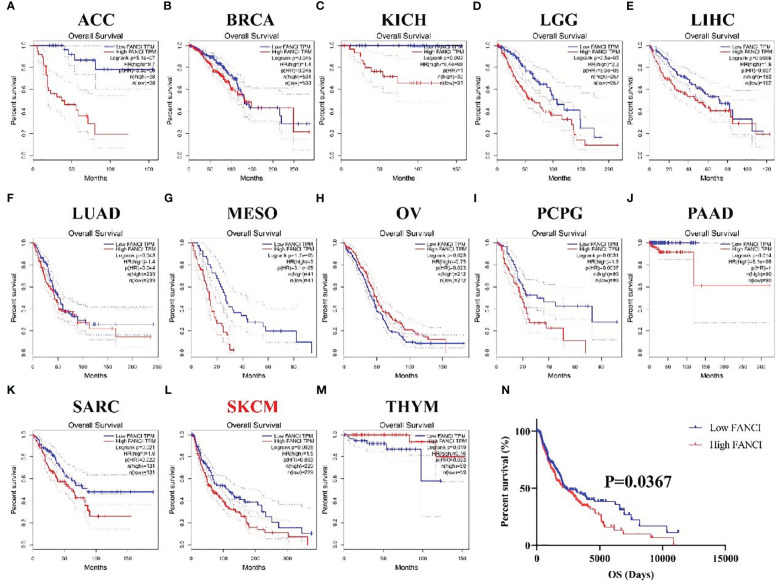
The prognosis values of FANCI in multiple tumors. The OS in patients with differential FANCI in ACC **(A)**, BRCA **(B)**, KICH **(C)**, LGG **(D)**, LIHC **(E)**, LUAD **(F)**, MESO **(G)**, OV **(H)**, PCPG **(I)**, PAAD **(J)**, SARC **(K)**, SKCM **(L)**, and THYM **(M)** from the GEPIA database. The OS in patients with SKCM with differential FANCI based on TCGA-SKCM datasets **(N)**.

We examined the connection between FANCI expression and the prognosis in 33 diverse cancers using the GEPIA database to explore the prognostic value of FANCI in different cancers. The higher FANCI expression has worse OS in ACC, BRCA, KICH, LGG, LIHC, LUAD, MESO, PCPG, PAAD, SARC, and SKCM (**Figures 2A–G**, **2I–2L**). Low FANCI expression was also correlated with decreased OS in OV (**Figure 2H**) and THYM (**Figure 2M**). No different prognostic value was found between higher FANCI and lower FANCI in BLCA, CESC, CHOL, COAD, DLBC, ESCA, GBM, HNSC, KIRC, KIRP, LAML, LUSC, PRAD, READ, STAD, TGCT, THCA, UCEC, UCS, and UVM (**Supplementary Figures 2A–T**). High FANCI expression predicted a worse prognosis for SKCM patients based on the TCGA-SKCM datasets (**Figure 2N**). To sum up, FANCI can be a biomarker for SKCM prognosis.

### 3.3 FANCI regulates the cytokines in SKCM

**Figure 3:**
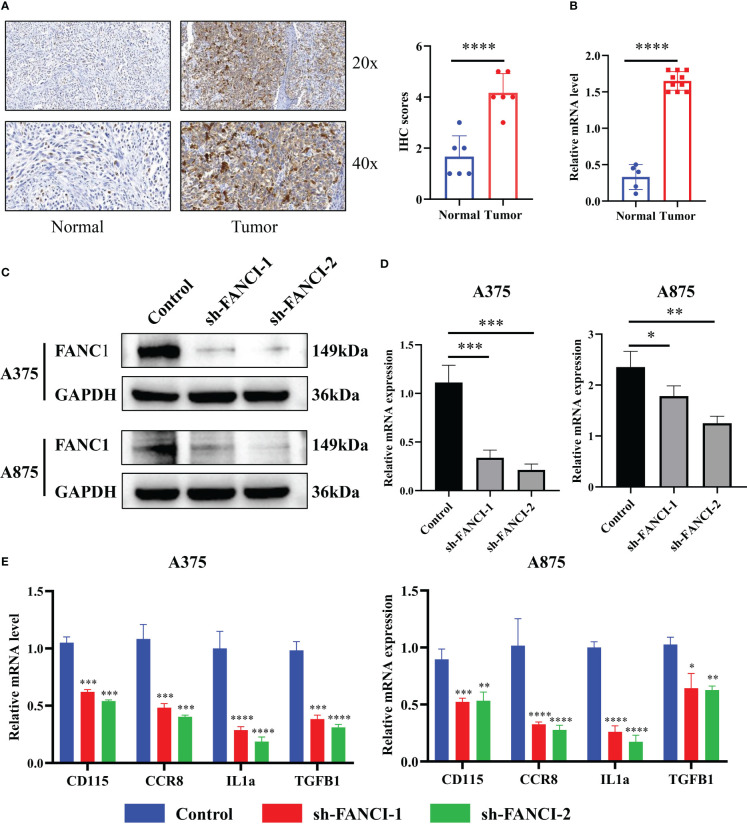
FANCI regulates the cytokines in SKCM. **(A)** Representative picture of FANCI expression level from tumor and normal tissues by IHC. **(B)** The mRNA expression of FANCI in tumor and normal tissues by RT-qPCR analysis. **(C)** FANCI knockdown in A375 and A875 cell lines. **(D)** RT-qPCR was used to detect the knockdown efficiency of FANCI in A375 and A875. **(E)** The expression of CD115, CCR8, IL1α, and TGFB1 in A375 and A875 by RT-qPCR. *P < 0.05, **P < 0.01, ***P < 0.001, ****P < 0.0001.

We conducted IHC staining on tumor tissues in SKCM patients and normal skin tissues to further explore the mechanism of FANCI in SKCM. The result revealed that FANCI was upregulated in SKCM tissues (**Figure 3A**). Consistently, the mRNA level of FANCI was increased in SKCM compared to normal skin tissues (**Figure 3B**). The results from the TCGA-SKCM and GEO datasets were comparable with the IHC and RT-qPCR. Bioinformatics analysis and our own data results both indicate the involvement of FANCI as an oncogene in SKCM. We used lentiviral transduction technology to knock down FANCI expression in A375 and A875 cell lines (sh-FANCI-1 and sh-FANCI-2) to further explore the oncogenic role of FANCI in SKCM pathogenesis, and the Western blot experiment demonstrated that we successfully established FANCI knockdown cell lines (**Figure 3C**). The RT-qPCR results similarly illustrate that sh-FANCI-1 and sh-FANCI-2 exhibit greater transfection efficiency in A375 and A875 cells (**Figure 3D**). We then detected the expression of cytokines, such as cluster of differentiation (CD) 115, CCR8, interleukin (IL) 1α, and transforming growth factor beta-1 (TGFB1), in A375 and A875 cell lines with FANCI knockdown expression. **Figure 3E** shows that FANCI knockdown downregulated CD115, CCR8, IL1α, and TGFB1 expression both in A375 and A875 cells, which indicated that FANCI knockdown places SKCM in an inhibitory state against cytokines, which greatly benefits SKCM treatment.

### 3.4 FANCI plays as an oncogene in SKCM

**Figure 4:**
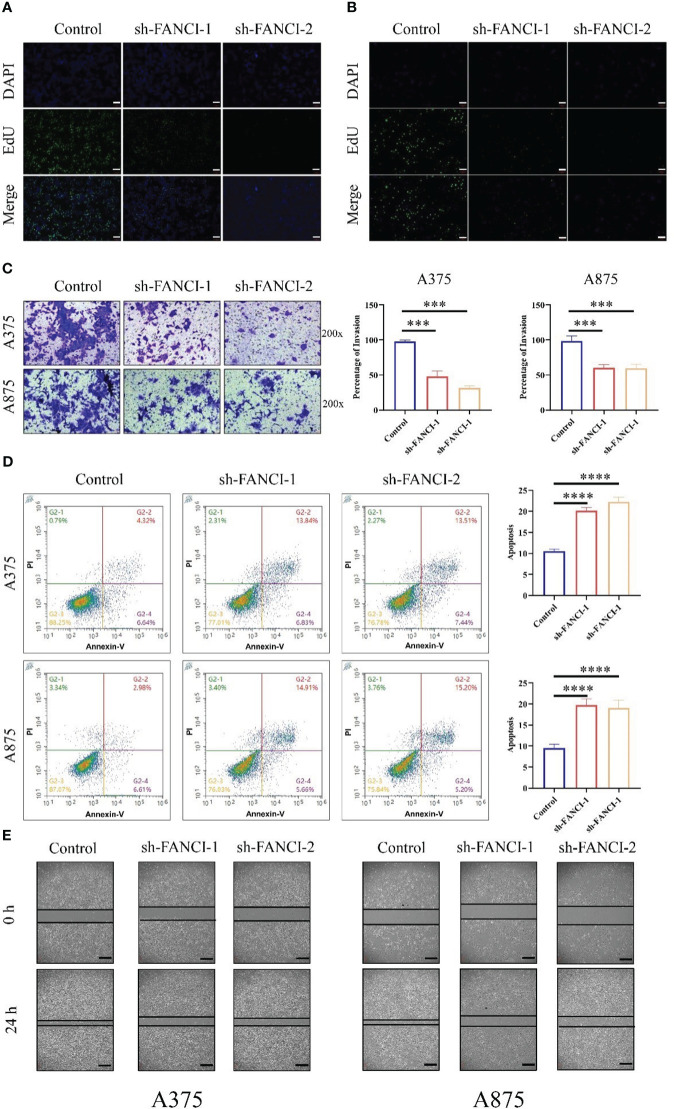
FANCI plays as an oncogene in SKCM. The proliferation ability of A375 **(A)** and A875 **(B)** cells was evaluated using EdU after knocking down FANCI and control, scale bar: 100μm. **(C)** Representative cell invasion in A375 and A875 cells after knocking down FANCI and control. **(D)** Representative cell apoptosis in A375 and A875 cells after knocking down FANCI and control. **(E)** Wound healing assay in each group, scale bar: 200μm. ***P < 0.001, ****P < 0.0001.

Subsequently, we further explored the regulatory role of FANCI on SKCM. EdU showed that FANCI downregulation suppressed the growth of A375 (**Figure 4A**) and A875 (**Figure 4B**). Compared with the control group, FANCI knockdown significantly decreased the invasive ability of A375 and A875 cells (**Figure 4C**). Undoubtedly, knocking down FANCI expression can significantly promote the apoptotic ability of A375 and A875 cells (**Figure 4D**). Consistent with the results of the transwell assays, the cell scratch assay revealed that the migration rate of cells with FANCI knockdown decreased within 24 h (**Figure 4E**). In conclusion, FANCI knockdown in A375 and A875 cell lines not only inhibits the malignant phenotypes of SKCM cells but also reduces the cytokines (CD115, CCR8, IL1α, and TGFB1). These findings indicate that FANCI plays multiple roles in SKCM, expanding its oncogenic effects to include cytokine regulation and biology representation.

### 3.5 FANCI may regulate immune microenvironment of SKCM

**Figure 5:**
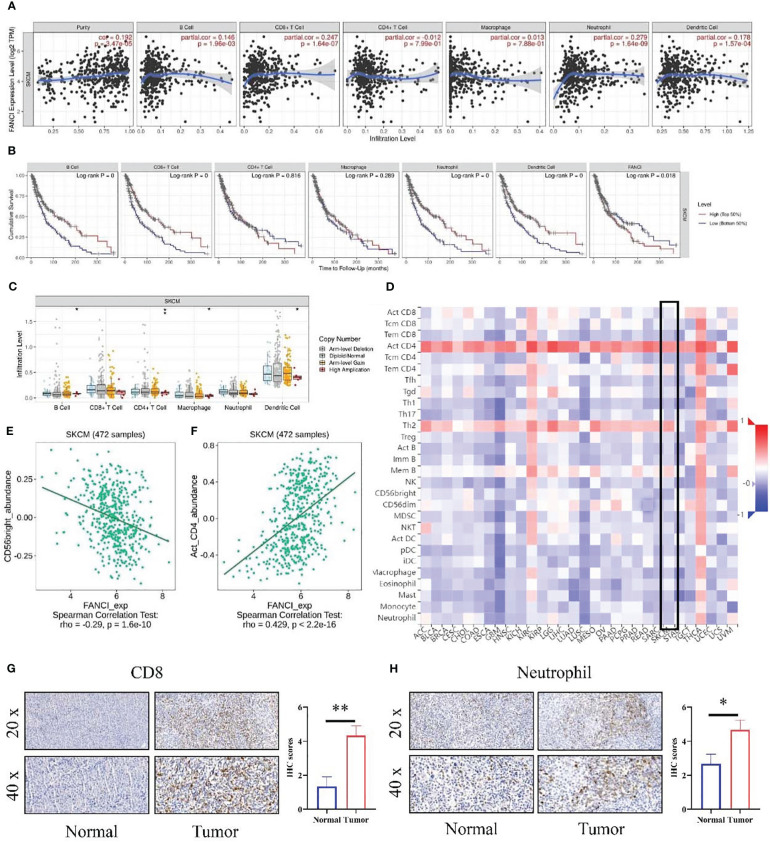
The relationship between the level of FANCI and immune infiltration from SKCM. **(A)** FANCI was correlated with immune cell infiltration in SKCM. **(B)** K-M plots in SKCM. **(C)** The correlation of SCNA of FANCI and immune infiltration. **(D)** The correlation between FANCI and 28 immune cells from the TISIDB database. **(E)** FANCI was negatively correlated with CD56bright cells. **(F)** FANCI was positively correlated with neutrophils. **(G)** IHC detected the difference expression of CD8 between normal and tumor tissues. **(H)** The difference of neutrophil between normal and tumor tissues was determined by IHC. *P < 0.05, **P < 0.01.

The tumor immune microenvironment dysfunction may accelerate tumor development and progression. Therefore, we investigated the potential involvement of FANCI in the immune microenvironment of SKCM. Our data indicated that the expression level of FANCI was positively correlated with the infiltration degree of different infiltrating immune cell types using the TIMER database, including neutrophils (R = 0.279, P = 1.64e-9), CD8+ T cells (R = 0.247, P = 1.64e-7), dendritic cells (R = 0.178, P = 1.57e-4), and B cells (R = 0.146, P = 1.96e-3) (**Figure 5A**). FANCI and CD4+ T cells or macrophage cells demonstrated no apparent correlation. We generated Kaplan–Meier curves using the TIMER database to display immune cell infiltration and FANCI expression in SKCM. Neutrophils, CD8+ T cells, dendritic cells, and B cells were significantly correlated with SKCM survival (**Figure 5B**). The high expression of immune cells, including neutrophils, CD8+ T cells, dendritic cells, and B cells, signifies a favorable prognosis for patients with patients SKCM. Whereas, the unfavorable prognosis is shown when FANCI is highly expressed. In summary, these experimental data indicate the involvement of FANCI in regulating immune cell infiltration in SKCM and are associated with the clinical outcomes of SKCM in collaboration with some immune cells. Moreover, FANCI showed significant correlations with infiltration degrees of immune cells (**Figure 5C**). We exploited the TISIDB to explore the relationship between FANCI level and the abundance of 28 types of TILs (**Figure 3C**), which is consistent with the aforementioned results (**Figure 5D**). **Supplementary Table 1** shows that FANCI expression is negatively correlated with the abundance of most tumor-infiltrating cells. CD56bright natural killer cell (**Figure 5E**) or activated CD4 T cell (**Figure 5F**) showed the top significant negative or positive correlation. The results confirmed that CD8 and neutrophils were promoted in SKCM tissues by IHC (**Figures 5G, H**).

### 3.6 High expression of FANCI suppresses immune infiltration

**Figure 6:**
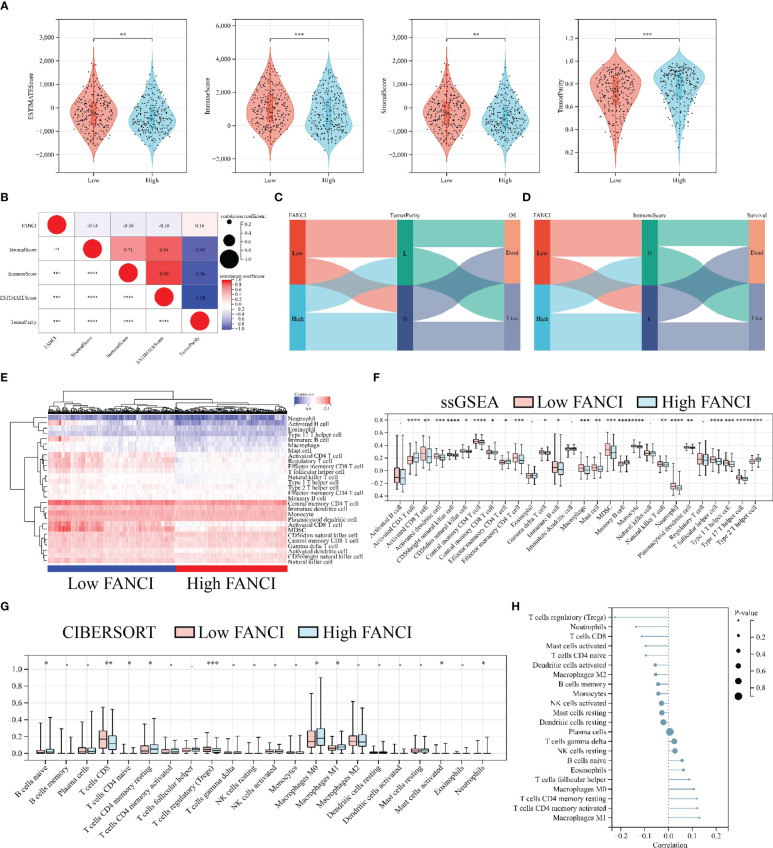
High FANCI expression suppresses immune infiltration. **(A)** The expression of ESTIMATEScore, ImmuneScore, StromalScore, and TuomrPurity between the higher and the lower FANCI groups. **(B)** The correlation of FANCI and ESTIMATEScore, ImmuneScore, StromalScore, and TuomrPurity. **(C)** The Sankey diagram illustrates the correlation between FANCI, TuomrPurity, and survival status. **(D)** The Sankey diagram illustrates the correlation between FANCI, ImmuneScore, and survival status. **(E)** The heatmap displays the expression of 28 TIICs in each patient in TCGA-SKCM. **(F)** The expression of 28 TIICs between the higher and the lower FANCI groups. **(G)** The expression of 22 immune cells between the higher and the lower FANCI groups. **(H)** The correlation of FANCI and 22 immune cells. *P < 0.05, **P < 0.01, ***P < 0.001, ****P < 0.0001.

We performed a series of analyses to further explore the impact of FANCI on the tumor immune microenvironment in SKCM patients. The results from the ESTIMATE algorithm revealed a significantly downregulated ImmuneScore in the higher FANCI group; however, the TumorPurity was upregulated in the higher FANCI group versus the lower FANCI group (**Figure 6A**). The FANCI expression is negatively correlated with ImmuneScore, StromalScore, and EstimateScore, while positively correlated with TumorPurity (**Figure 6B**). Patients with increased FANCI expression in SKCM exhibit elevated TumorPurity expression and experience a correspondingly higher mortality rate. Conversely, patients with heightened FANCI expression in SKCM demonstrate reduced ImmuneScore expression and have a higher mortality rate (**Figures 6C, D**). Additionally, most TIICs were upregulated in the higher FANCI group (**Figures 6E, F**). CIBERSORT algorithm analysis revealed increased the CD8 and CD4 expression of T cells in the lower FANCI group and significantly decreased M0 and M1 macrophage expressions in the lower FANCI group (**Figure 6G**). FANCI expression showed a negative correlation with T cells regulatory (Tregs), neutrophils, T cells CD8, mast cells activated, and T cells CD4 naive. However, it demonstrated a positive correlation with M0 macrophage, T cells CD4 memory resting, T cells CD4 memory activated, and M1 macrophage (**Figure 6H**). These results indicate that FANCI upregulation results in an immunosuppressive state in patients with SKCM, as well as an increase in M1 macrophage polarization, thereby increasing inflammation.

### 3.7 FANCI can predict immunotherapy responses of SKCM

**Figure 7:**
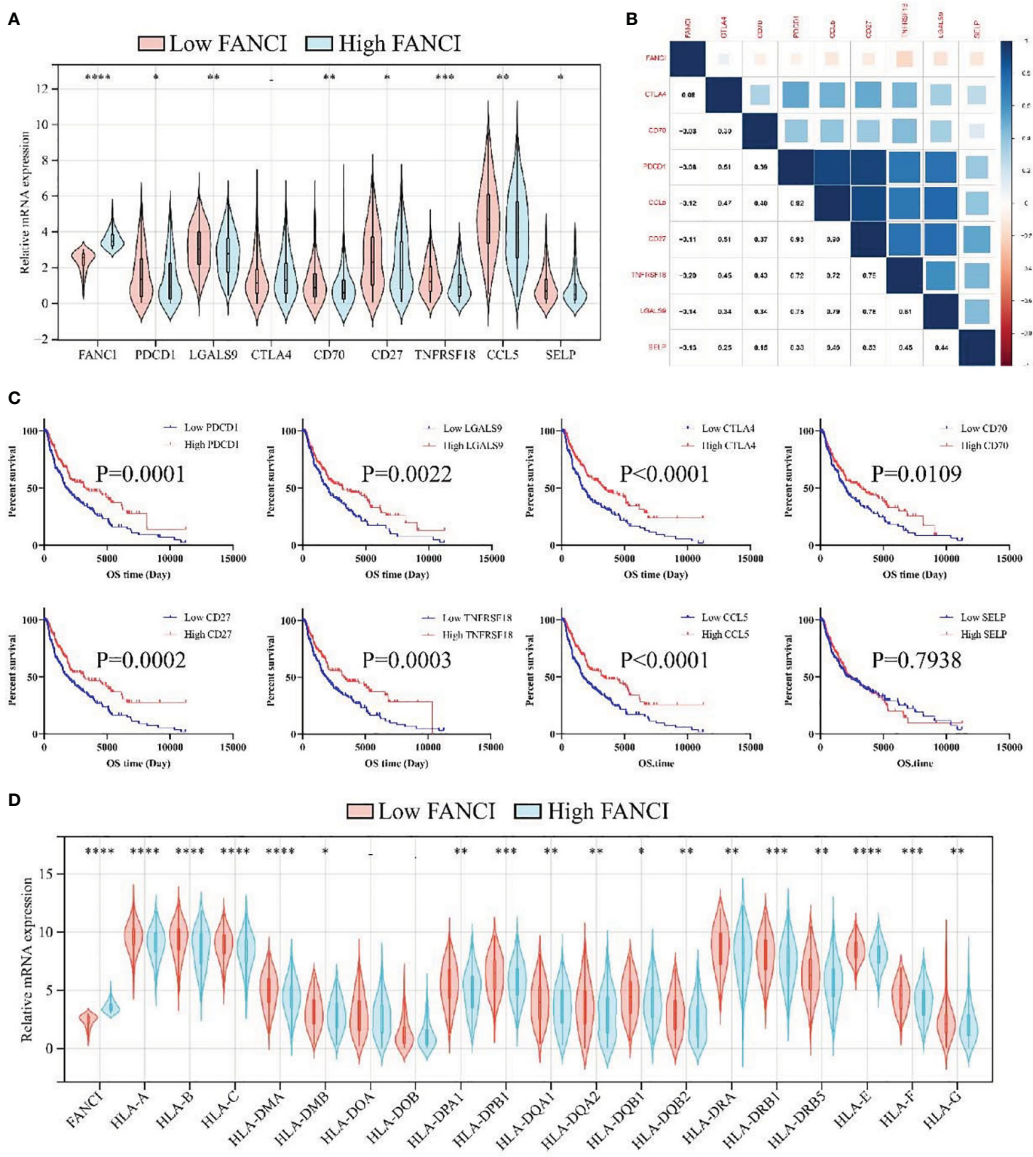
FANCI can predict immunotherapy responses of SKCM. **(A)** The expression of eight immune checkpoints between the higher and the lower FANCI groups. **(B)** The correlation of FANCI and immune checkpoint. **(C)** The OS in patients with SKCM with differential immune checkpoint based on the TCGA-SKCM datasets. **(D)** The expression of MHC genes between the higher and the lower FANCI groups. *P < 0.05, **P < 0.01, ***P < 0.001, ****P < 0.0001.

The previous results indicate that FANCI regulates the immune microenvironment of SKCM, and we speculate that FANCI is associated with the immune therapeutic response of SKCM. FANCI expression level is significantly reduced in individuals who respond to immunotherapy, and the expression level of their immune checkpoint is significantly higher than that of individuals who do not respond to immunotherapy (**Figure 7A**). FANCI expression and the majority of immune checkpoints showed a negative correlation, while patients with SKCM with high immune checkpoints demonstrated a favorable prognosis, which further highlights the reason why patients with SKCM with high FANCI expression experience a poor prognosis (**Figures 7B, C**). The lower FANCI group and the stable antigen presentation function demonstrated a notable correlation, as evidenced by the frequently elevated levels of major histocompatibility complex (MHC) expression in this group compared to the higher FANCI group (**Figure 7D**). In general, SKCM patients with lower FANCI expression tend to show more advantages from immunotherapy than patients of high FANCI expression. This enables the development of a customized treatment strategy for patients with SCKM.

## 4 Discussion

Melanoma, a cancer that can develop directly from melanocytes, is known for its invasiveness and destructiveness (27). Researchers conducted extensive studies on the pathogenesis and treatment methods of SKCM in recent years. The primary factor that causes a high mortality rate in melanoma is its strong ability to metastasize. The most effective method to treat melanoma remains the surgical tumor removal before it metastasizes (28). Treating metastatic SKCM was difficult and unsatisfactory before the introduction of immunotherapy (29, 30). Additionally, the 5-year survival rate of these patients tends to have significant increase due to immunotherapy (31). However, the immunotherapy is limited due to drug resistance and ineffectiveness (32). A previous study revealed that the average objective response rate of anti-CTLA-4 antibodies was 14%, while that of anti-PD-1 monoclonal antibodies was 33% (33). Therefore, highly effective biomarkers and immunotherapeutic targets are urgently required for diagnosis and treatment.

This study identified 1829 DEGs between SKCM and healthy skin tissues from the GSE46517 dataset, in which FANCI was significantly upregulated in SKCM tissues. Additionally, the SKCM-TCGA, GSE15605, and GSE114445 datasets also demonstrate a significant FANCI expression upregulation in SKCM tumor tissues. FANCI significantly upregulated in SKCM tissues through IHC and RT-qPCR detection, thereby confirming the accuracy of the bioinformatics results. Moreover, the patients with higher FANCI have a worse prognosis. An imbalanced FANCI has been indicated to be significant in SKCM development. Additionally, FANCI knockdown suppressed inflammatory factors such as CD115, CCR8, IL1α, and TGFB1. Furthermore, the reduced FANCI can inhibit the malignant abilities in SKCM cells.

It is reported that FANCI is overexpressed in numerous tumors. The high FANCI expression level may boost up tumor growth in lung adenocarcinoma by inhibiting M1 macrophages (34, 35). Additionally, FANCI expression in liver hepatocellular carcinoma showed a positive relationship with tumor infiltration levels (36). These results indicated that FANCI may regulate the immune microenvironment of tumors. Immune cell infiltration into tumor tissues can modify the metabolism and functioning, which can enhance immune suppression and immune evasion (37). The researchers found that the presence of T cells, B cells, and mature dendritic cells within tumor tissues was associated with an extended survival period in patients with SKCM (38). The elevated CD8+ T cells and Treg cells in different cancer types has been related to positive clinical outcomes (38). Our results demonstrated that high infiltration of B cells, CD8+ T cells, neutrophils, and dendritic cells showed better outcomes, which indicated that treating SKCM through immunotherapy may be an acceptable approach.

The tumor microenvironment comprises noncancerous cells, with a significant influence on tumor genomic analysis (39). The TIICs recognize and advance tumor cell apoptosis, and thus suppress tumor progression. Simultaneously, immune cells may select tumor cells that are better adapted to survive in the microenvironment, thereby facilitating tumor advancements (40). Our study revealed that immune infiltration analysis indicates that FANCI may be closely associated with immune response regulation and immune cells, particularly CD8+ T cells, dendritic cells, and B cells. CD8+ T and natural killer (NK) cells use various mechanisms to induce cell death and thwart tumor cells from proliferating (41). Additionally, activated B cells may generate antibodies targeted at tumor cells, boost the activity of CTLs, or release granule enzyme B to directly eliminate tumor cells (42). Interestingly, excessive FANCI expression may increase the levels of cellular immune suppression. FANCI also enhanced the infiltration and functioning of immunosuppressive cells using certain pathway downstream. Hence, these immunosuppressive cells work together to limit the growth and activation of CD8+ T cells, and the infiltration of NK cells (43, 44).

## 5 Conclusion

This study revealed that FANCI expression was upregulated in SKCM and was associated with SKCM metastasis. We also explored FANCI’ potential as a prognostic and therapeutical biomarker in SKCM. Additionally, FANCI had a positive correlation with immune infiltration, which indicated that FANCI may affect the immune microenvironment of SKCM and function as a favorable immunotherapeutic target for SKCM. Nevertheless, further investigation is needed to explore the regulatory role of FANCI in SKCM due to the existing limitations in our article.

## Data availability statement

The datasets presented in this study can be found in online repositories. The names of the repository/repositories and accession number(s) can be found in the article/**Supplementary Material**.

## Ethics statement

The studies involving humans were approved by Ethics Committee of Shuguang Hospital of Shanghai University of Traditional Chinese Medicine. The studies were conducted in accordance with the local legislation and institutional requirements. The participants provided their written informed consent to participate in this study. Written informed consent was obtained from the individual(s) for the publication of any potentially identifiable images or data included in this article.

## Author contributions

ZC: Conceptualization, Formal Analysis, Investigation, Methodology, Validation, Writing – original draft, Writing – review and editing. YD: Conceptualization, Funding acquisition, Methodology, Project administration, Writing – original draft, Writing – review and editing. WL: Formal Analysis, Project administration, Validation, Writing – review and editing. ZL: Methodology, Project administration, Writing – review and editing. ZG: Project administration, Validation, Writing – review and editing. XH: Project administration, Validation, Writing – review and editing. XX: Data curation, Project administration, Writing – review and editing. YC: Methodology, Project administration, Writing – review and editing. XB: Conceptualization, Data curation, Formal Analysis, Supervision, Validation, Writing – original draft, Writing – review and editing. WW: Conceptualization, Data curation, Funding acquisition, Project administration, Validation, Writing – original draft, Writing – review and editing. SH: Formal Analysis, Project administration, Writing – review and editing.

## Supplementary material

Supplementary Figure 1 DEGs between SKCM and normal tissues. **(A)** The volcano plot demonstrates DEGs between SKCM and normal tissues. **(B)** The histogram demonstrates DEGs between SKCM and normal tissues.

Supplementary Figure 2 The prognosis values of FANCI in multiple tumors. The OS in patients with differential FANCI in BLCA **(A)**, CESC **(B)**, CHOL **(C)**, COAD **(D)**, DLBC **(E)**, ESCA **(F)**, GBM **(G)**, HNSC **(H)**, KIRC **(I)**, KIRP **(J)**, LAML **(K)**, LUSC **(L)**, PRAD **(M)**, READ **(N)**, STAD **(O)**, TGCT **(P)**, THCA **(Q)**, UCEC **(R)**, UCS **(R)**, and UVM **(T)** from the GEPIA database.
