# Supplementary figures and images for: FANCI serve as a prognostic biomarker correlated with immune infiltrates in skin cutaneous melanoma
Source: Front Immunol. 2023 Nov 22;14:1295831. doi: 10.3389/fimmu.2023.1295831 (PMC10703153; doi:10.3389/fimmu.2023.1295831)

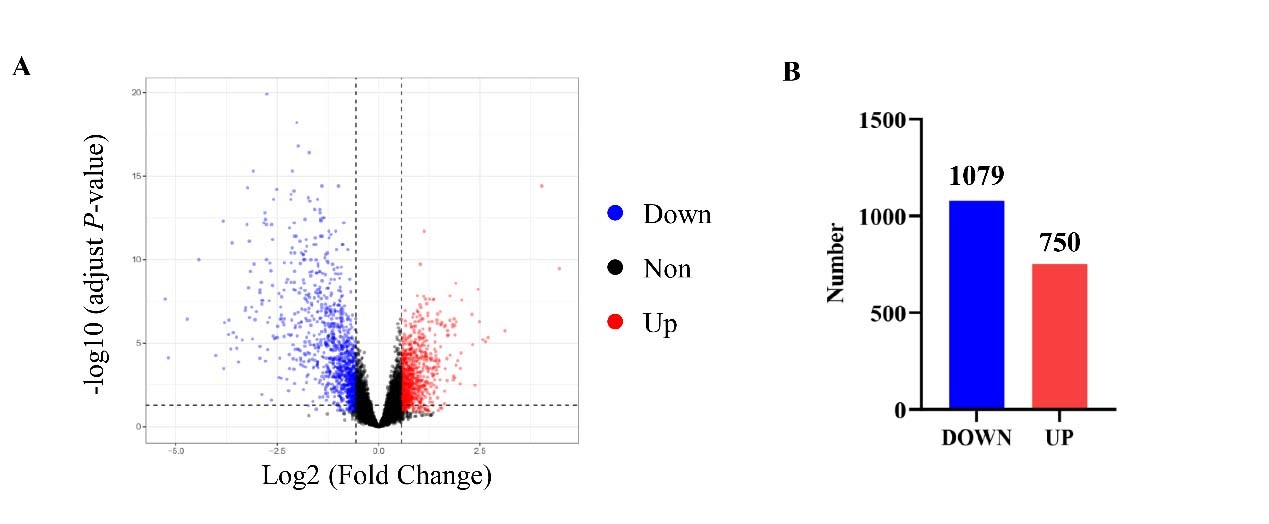

Supplement: Supplementary Figure 1 — DEGs between SKCM and normal tissues. (A) The volcano plot demonstrates DEGs between SKCM and normal tissues. (B) The histogram demonstrates DEGs between SKCM and normal tissues. [file Image_1.jpeg]

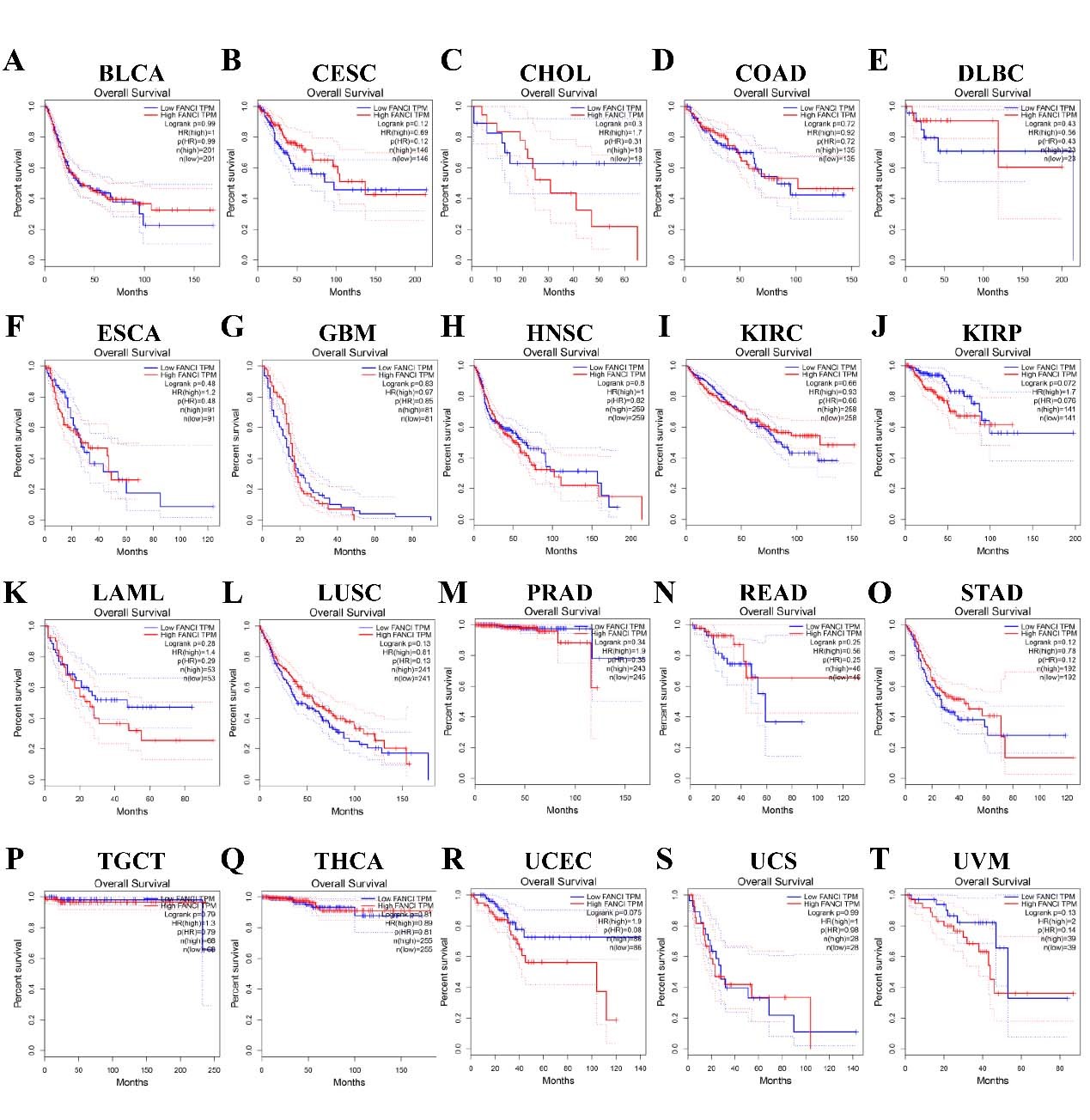

Supplement: Supplementary Figure 2 — The prognosis values of FANCI in multiple tumors. The OS in patients with differential FANCI in BLCA (A), CESC (B), CHOL (C), COAD (D), DLBC (E), ESCA (F), GBM (G), HNSC (H), KIRC (I), KIRP (J), LAML (K), LUSC (L), PRAD (M), READ (N), STAD (O), TGCT (P), THCA (Q), UCEC (R), UCS (R), and UVM (T) from the GEPIA database. [file Image_2.jpeg]
