# Supplementary material for: FANCI serve as a prognostic biomarker correlated with immune infiltrates in skin cutaneous melanoma
Source: Front Immunol. 2023 Nov 22;14:1295831. doi: 10.3389/fimmu.2023.1295831 (PMC10703153; doi:10.3389/fimmu.2023.1295831)
Supplement: Supplementary file 3 [file Table_1.docx]

| Cell Name | Spearman Correlation  Test: Rho | P-value |
| --- | --- | --- |
| Activated CD8 T cell | -0.106 | 0.0216 |
| Central memory CD8 T cell | -0.146 | 0.00153 |
| Effector memory CD8 T cell | -0.243 | 1.04e-7 |
| Activated CD4 T cell | 0.429 | <2.2e-16 |
| Central memory CD4 T cell | -0.284 | 3.99e-10 |
| Effector memory CD4 T cell | 0.129 | 0.00497 |
| T follicular helper cell | -0.225 | 8.84e-07 |
| Gamma delta T cell | -0.128 | 0.00543 |
| Type 1 T helper cell | -0.246 | 6.85e-08 |
| Type 17 T helper cell | -0.228 | 6.2e-07 |
| Type 2 T helper cell | 0.358 | 1.18e-15 |
| Regulatory T cell | -0.147 | 0.00133 |
| Activated B cell | -0.142 | 0.00203 |
| Immature B cell | -0.163 | 0.000377 |
| Memory B cell | 0.173 | 0.000159 |
| Natural killer cell | -0.143 | 0.00186 |
| CD56bright natural killer cell | -0.29 | 1.6e-10 |
| CD56dim natural killer cell | -0.138 | 0.00271 |
| Myeloid derived suppressor cell | -0.23 | 4.97e-07 |
| Natural killer T cell | -0.125 | 0.0064 |
| Activated dendritic cell | -0.188 | 3.89e-05 |
| Plasmacytoid dendritic cell | -0.186 | 4.83e-05 |
| Immature dendritic cell | -0.048 | 0.302 |
| Macrophage | -0.2 | 1.29e-05 |
| Eosinophil | -0.067 | 0.143 |
| Mast cell | -0.201 | 1.11e-05 |
| Monocyte | -0.243 | 1.03e-07 |
| Neutrophil | -0.232 | 3.63e-07 |

Table 1 Association Between the Expression of FANCI and the Abundance of Tumor-Infiltrating Lymphocytes.
